# Pilocytic astrocytoma: pathology, molecular mechanisms and markers

**DOI:** 10.1007/s00401-015-1410-7

**Published:** 2015-03-20

**Authors:** V. Peter Collins, David T. W. Jones, Caterina Giannini

**Affiliations:** 1 Department of Pathology, Addenbrooke’s Hospital, University of Cambridge, Cambridge, UK; 2 Division of Pediatric Neurooncology, German Cancer Research Center (DKFZ), Heidelberg, Germany; 3 Department of Laboratory Medicine and Pathology, Mayo Clinic, Rochester, MN USA; 4 Department of Histopathology, Addenbrooke’s Hospital, University of Cambridge, Box 235, Hills Road, Cambridge, CB2 2QQ England, UK

**Keywords:** Pilocytic astrocytoma, Brain neoplasms, Histopathology, Morphology, Immunocytochemistry, Oncogenes, Molecular pathology, MAPK

## Abstract

Pilocytic astrocytomas (PAs) were recognized as a discrete clinical entity over 70 years ago. They are relatively benign (WHO grade I) and have, as a group, a 10-year survival of over 90 %. Many require merely surgical removal and only very infrequently do they progress to more malignant gliomas. While most show classical morphology, they may present a spectrum of morphological patterns, and there are difficult cases that show similarities to other gliomas, some of which are malignant and require aggressive treatment. Until recently, almost nothing was known about the molecular mechanisms involved in their development. The use of high-throughput sequencing techniques interrogating the whole genome has shown that single abnormalities of the mitogen-activating protein kinase (MAPK) pathway are exclusively found in almost all cases, indicating that PA represents a one-pathway disease. The most common mechanism is a tandem duplication of a ≈2 Mb-fragment of #7q, giving rise to a fusion between two genes, resulting in a transforming fusion protein, consisting of the N-terminus of KIAA1549 and the kinase domain of BRAF. Additional infrequent fusion partners have been identified, along with other abnormalities of the MAP-K pathway, affecting tyrosine kinase growth factor receptors at the cell surface (e.g., FGFR1) as well as BRAF V600E, KRAS, and NF1 mutations among others. However, while the KIAA1549-BRAF fusion occurs in all areas, the incidence of the various other mutations identified differs in PAs that develop in different regions of the brain. Unfortunately, from a diagnostic standpoint, almost all mutations found have been reported in other brain tumor types, although some retain considerable utility. These molecular abnormalities will be reviewed, and the difficulties in their potential use in supporting a diagnosis of PA, when the histopathological findings are equivocal or in the choice of individualized therapy, will be discussed.

## Introduction

The term “pilocytic” to describe astrocytoma variants has been used since the 1930s [8, 18] to indicate cells with hair-like, bipolar processes. Today, what we call pilocytic astrocytoma (PA) has had a number of names before the WHO Classification System became generally accepted; older terms include “polar spongioblastoma” and “juvenile astrocytoma”. The importance of distinguishing the relatively benign PA from the other more aggressive “diffuse gliomas” has been recognized by many authors for at least 70 years [8]. Despite the worldwide acceptance of the WHO Classification by neuropathologists, these tumors are still clinically referred to by a number of terms, including cerebellar astrocytoma, optic glioma, and infundibuloma, because of the distinct predilection for young patients and certain anatomic sites, including the cerebellum, optic pathways, and third ventricular/hypothalamic region.

In this paper, we will review our current knowledge of the histopathological and molecular aspects of PA. As defined in the current WHO Classification System [42], PA makes up approximately 5.1 % of all gliomas and is most common in children [47]. Males are slightly more frequently affected than females. According to the CBTRUS statistical report [48], PA is the most frequent primary brain tumor in 0- to 19-year olds, with an average annual age-adjusted incidence rate (adjusted to the 2000 US standard population) of 0.84 (per 100,000), which substantially declines from the 10–14 years age group to the 15–19 years age group. Pilocytic astrocytoma accounts for 15.4 % of children and adolescents (019 years) and 17.6 % of childhood (0–14 years) primary brain tumors. Other studies indicate an incidence rate of 4.8 per 1 million per year [9]. PA, however, may occur at any age, becoming increasingly uncommon with advancing years [48]. PA can arise anywhere in the CNS, although it most frequently occurs in the cerebellum (42 %), followed by the supratentorial compartment (36 %), the optic pathway and hypothalamus (9 %), brainstem (9 %), and the spinal cord (2 %) [9]. In children, the most common site affected is the cerebellum (67 %), with only rare cases developing supratentorially; while in adults, there was no significant difference between cerebellum and supratentorial compartment (33 % each) [9].

Almost all PAs are considered WHO grade I. A rare variant termed “pilomyxoid astrocytoma,” occurring predominantly in children under 1 year of age and in the hypothalamic/chiasmatic region, has been assigned WHO grade II. Uncommonly, cellular and highly atypical PAs with frequent mitoses may be seen and could be considered on the basis of the histological findings to be anaplastic, but no WHO grade III variant is included in the WHO Classification. Both these rare variants will be discussed below. For a short review of the WHO grading system, see [42, 54].

## Clinical symptoms

Presenting symptoms will generally be insidious due to the slow growth of the tumor, and the identification of early symptoms will be dependent on localization and the ability of the patient to communicate neurological change and discomfort resulting from, for example, increased intracranial pressure. Common presenting symptoms for cerebellar tumors include ataxia, cranial nerve defects, and signs of increased intracranial pressure (headache, nausea and vomiting). When present in the optic pathways, the tumors may produce loss of visual acuity or field defects and, when localized to the hypothalamus, may result in endocrine syndromes, such as diabetes insipidus, precocious puberty, or electrolyte imbalance. Blocking of CSF pathways may result in hydrocephalus with rapid deterioration.

## Neuroimaging

**Fig. 1:**
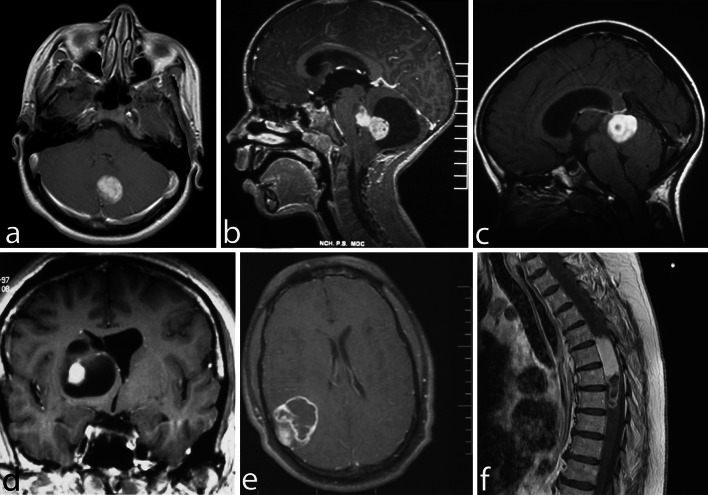
Pilocytic astrocytoma, with its characteristic imaging features, may occur virtually at any site in the CNS. Six different examples (all histologically confirmed) with strong contrast enhancement are illustrated: two cerebellar examples, one of a small left para-vermian well-circumscribed and solid tumor (**a**) and one of a cystic tumor with a mural nodule (**b**); a “dorsally exophytic” midbrain PA (**c**); a cyst with a mural nodule occupying the right thalamus (**d**); a peripheral solid and cystic tumor in the right parietal lobe (**e**); and a large, circumscribed, intramedullary, tumor with a cystic component (**f**)

**Fig. 2:**
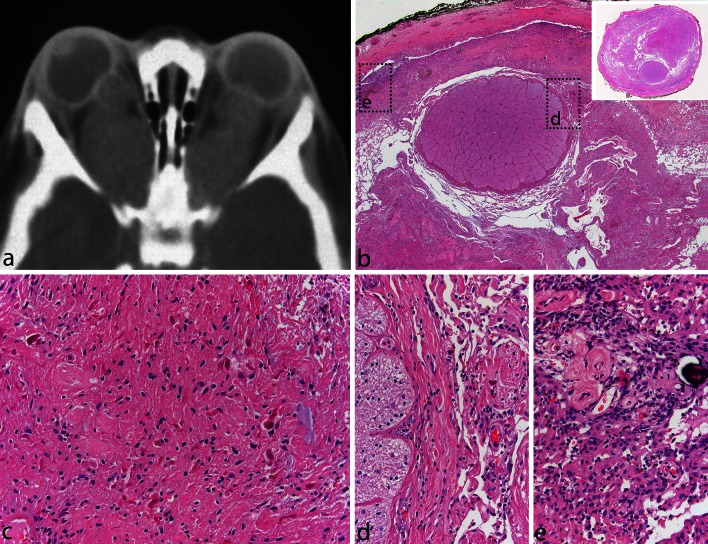
Pilocytic astrocytoma of the optic nerve. Bilateral fusiform enlargement of the optic nerve is virtually diagnostic of neurofibromatosis type 1 (**a**). The tumor typically extends into the leptomeningeal space, expanding the dural sheath and compressing the remaining optic nerve proper, which is atrophic (**b**). A complete cross section of the optic nerve is shown in the *inset*. The tumor has classic PA features with a densely fibrillated appearance and numerous Rosenthal fibers (**c**). The interface between the optic nerve and the tumor is shown in (**d**), while the subdural region shows meningothelial hyperplasia, at times, with scant psammoma bodies (**e**)

Pilocytic astrocytomas usually appear on CT scans as round/oval lesions that are well-defined iso- or slightly hypo-dense and markedly enhance with contrast media. On MRI, PAs are typically hypo- or iso-intense on T1 sequences and hyperintense on T2-weighted or FLAIR images. They are typically strongly and diffusely enhancing (Fig. 1a–f). They may contain cysts or consist of a tumor nodule in a cyst (the latter being particularly common for cerebellar and hemispheric tumors) (Fig. 1b, d, e). Pilocytic astrocytoma, involving the optic pathways, optic nerve, and chiasm, typically form fusiform masses. It is the most common site in NF1 patients in whom bilateral tumors may arise (Fig. 2a). In the posterior fossa, PA may involve primarily the brainstem rather than the cerebellum. At this site, in contrast to diffuse intrinsic pontine gliomas, which infiltrate and expand primarily within the pons, PAs are generally located dorsally and have an exophytic pattern of growth (Fig. 1c) [20]. The spinal cord can also be affected (Fig. 1f) [44, 45].

## Pathology

Macroscopically, PAs are generally relatively soft in texture and gray in sections of fixed specimens. They appear to be well-defined. Cysts are common both within the tumor tissue as well as around the tumor, the latter resulting in a cyst with a tumor nodule (see Fig. 1b, d). Calcium deposits and hemosiderin may be present, the latter secondary to small bleeds into tumor tissue. Very rarely, PA can present with extensive leptomeningeal involvement without parenchymal involvement, the so-called “primary leptomeningeal gliomatosis” [6].

Histopathologically, PA is a tumor of low to moderate cellularity with compact, densely fibrillated areas rich in Rosenthal fibers, consisting of cells with long bipolar (hair-like) processes and elongated cytologically bland nuclei (Fig. 2c), as well as loosely textured areas, composed of multipolar cells (protoplasmic astrocyte-like), with bland, round-to-oval nuclei, and multiple, relatively short cytoplasmic extensions. These areas have varying degrees of mucoid background material with micro-cyst development being common, as are also eosinophilic granular bodies or hyaline droplets. The bipolar tumor cells are generally strongly GFAP immunoreactive, while the protoplasmic astrocyte-like tumor cells are less so. In some cases, areas morphologically similar to oligodendrogliomas may be found, but only rarely is the oligodendroglial-like component predominant (see “Differential diagnostic issues”). Cells with pleomorphic nuclei, often multinucleated, may also occur and generally are found in the loose microcystic regions. Rare mitoses are acceptable, but any notable mitotic activity should warrant the consideration of other glioma diagnoses. Ki67/MIB-1 indices of up to 4 % are common. Microvascular proliferation, resulting in relatively thick-walled, hyalinized, and/or glomeruloid vessels, is often seen, and infarct-like necrosis can occur in some cases (no pseudopalisading) [24]. While these findings are all compatible with a diagnosis of PA, they sometimes make the distinction from other gliomas difficult, particularly when examining small biopsies. While macroscopically appearing relatively well-defined, microscopically, varying degrees of invasion into the adjacent brain are observed [26]. Rare cerebellar tumors show a diffuse pattern of growth, and molecular analysis may be of some help in identifying these tumors as PAs (see below). Consequently, both normal astrocytes and neurons may become trapped in the tumor tissue. Microscopic infiltration of the leptomeninges frequently occurs, especially in the cerebellum and optic nerve tumors, and is not an ominous finding. Today, it is rare to see surgical resection specimens from optic nerve gliomas in NF1 patients, given the often benign and indolent natural history of these tumors, which may, at times, regress. On cross section, the optic nerve outline is often visible near the center of the specimen, while the tumor characteristically grows in the subarachnoid space between the nerve and the dural sheath that is markedly expanded (Fig. 2b–d). Meningothelial hyperplasia may occur and represent a potential pitfall in the differential diagnosis between optic nerve PA and optic nerve meningioma when only a small and superficial biopsy is obtained (Fig. 2e).

## Anaplasia in pilocytic astrocytoma

Most PAs are WHO grade I tumors and only rarely show histological features of anaplasia, e.g., hypercellularity, moderate to severe cytologic atypia in association with brisk mitotic activity, microvascular proliferation and/or necrosis (coagulative and/or pseudopalisading). Cases of apparent malignant transformation of a classic PA have been documented largely as case reports [1, 28, 36] and mainly following radiation therapy, but bona fide histologically malignant cases may occur in the absence of prior treatment. In a recent study of PAs, including a series of 34 PAs with anaplastic features, the frequency of anaplasia was very low (0.6 % among all PAs operated at the Mayo Clinic and 1.8 % among all consultation cases) [52]. Twenty-four of the 34 (71 %) PA with anaplastic features had a typical PA precursor, either coexistent (*n* = 14) (41 %) or documented by previous biopsy (*n* = 10); while the remaining 10 (29 %) exhibited typical pilocytic features in an otherwise anaplastic astrocytoma. Only four had received radiation. Eight patients (24 %) had a history of NF1. Four histological patterns of anaplasia were identified: (1) “pilocytic-like” (41 %) with classic bipolar cells with Rosenthal fibers and/or microcysts with eosinophilic granular bodies but with brisk mitotic activity and hypercellularity; (ii) poorly differentiated, small cell (32 %); (iii) epithelioid or rhabdoid (15 %); and (iv) cases resembling a classic diffusely infiltrative fibrillary astrocytoma (12 %). The presence of anaplastic features was associated with decreased survival when compared with typical PA. Interestingly, however, when compared to the historical cohort of diffusely infiltrative astrocytomas graded using the Mayo-St. Ann system [15], PA with anaplastic features grade-by-grade appeared to have a prognosis more favorable than their corresponding diffusely infiltrative astrocytomas. Unfortunately, due to the rarity of the PA with anaplasia cases and their largely consultative nature, no tissue was available for their further molecular characterization. Given the difficulty and subjectivity in making the diagnosis of PA with anaplastic features based solely on morphology, it is desirable that relevant molecular biomarkers, whenever available, are used to characterize prospectively these rare tumors and, in particular, to evaluate the presence of MAPK pathway alterations.

## Pilomyxoid astrocytoma variant

**Fig. 3:**
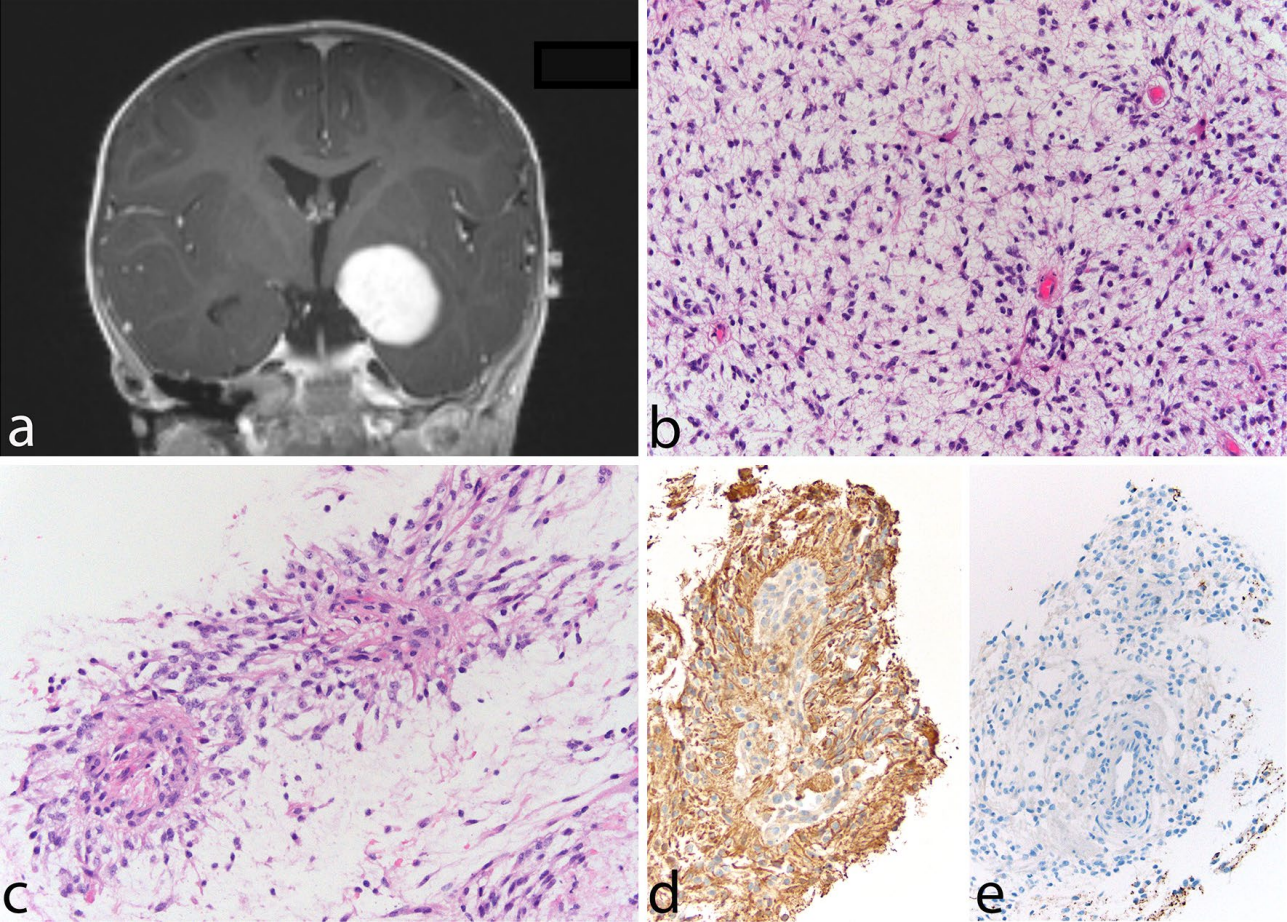
Pilomyxoid astrocytoma. A 13-month-old boy presented with a relatively circumscribed, strongly enhancing left medial temporal lobe mass (**a**). The tumor shows a monomorphous cell population in a loosely arranged myxoid background (**b**). Tumor cells with angiocentric arrangement and formation of pseudopapillary structures is typical (**c**). GFAP is typically positive in tumor cells (**d**), and immunocytochemistry for neurofilaments is negative, the tumor being generally relatively solid and devoid of axons (**e**)

Pilomyxoid astrocytoma (PMA) is a PA variant that occurs most commonly in the hypothalamic/chiasmatic region in very young children. The tumor was first reported in 1999 [63] and was included in the WHO Classification in 2007 when it was provisionally given a WHO malignancy grade of II [54]. It is typically composed of a monomorphous population of bipolar cells immersed in a myxoid/mucoid background, displaying a variable angiocentric arrangement with a tendency of the tumor to fall apart, at least focally, into pseudopapillae (Fig. 3a–e). Rosenthal fibers and eosinophilic granular bodies are characteristically absent. Pilomyxoid astrocytoma cells are typically strongly and diffusely positive for GFAP. Focal pilomyxoid features can be seen in otherwise classic PA and do not warrant the diagnosis of PMA. Some pilomyxoid tumors have been found to recur as classical PAs, suggesting that they may represent an early stage in the development of PAs [11, 31] and that their more sinister prognosis may be due to their identification in very young children and in regions where it is difficult to carry out a radical operation. As described below, some such cases have been shown to have the *KIAA1549*–*BRAF* fusion gene, which is common in sporadic PAs [14].

## Association with familial tumor syndromes

It has long been known that patients with neurofibromatosis type 1 (NF1) have an increased risk of all gliomas, with PA being the most frequent variant that occurs in about 15 % of these patients [40, 51]. The optic pathways are most commonly affected [39]. Until recently, NF1 patients were clinically diagnosed [19] with few undergoing mutation screening as the *NF1* gene spans 300 kb and is composed of 58 exons, with multiple different and complex mutations identified in the limited numbers of cases studied. With newer high throughput sequencing techniques, it will be interesting to see the full spectrum of mutations that occur and whether there is any particular association between distinct mutation types and the NF1 disease phenotype in the individual case as has been suggested by some studies [7, 57]. The NF1 protein, called neurofibromin, acts in the mitogen-activated protein (MAP) kinase pathway as a GTPase-activating protein for RAS, facilitating the deactivation of RAS. There is also an association between Noonan syndrome (a neuro-cardio-facial-cutaneous syndrome) characterized by germ-line mutations of MAP kinase pathway genes (*PTPN11*, *SOS1*, *KRAS*, *NRAS*, *RAF1*, *BRAF*, *SHOC2* and *CBL*) [3, 50] and PA (as well as other malignancies). The *PTPN11* gene is mutated in about 50 % of patients with Noonan syndrome and has been found also to be mutated (admittedly always together with *FGFR1* mutations, see below) in sporadic PAs [32]. However, the number of PAs reported in patients with Noonan syndrome is small [22, 46, 50].

## Molecular genetics

**Fig. 4:**
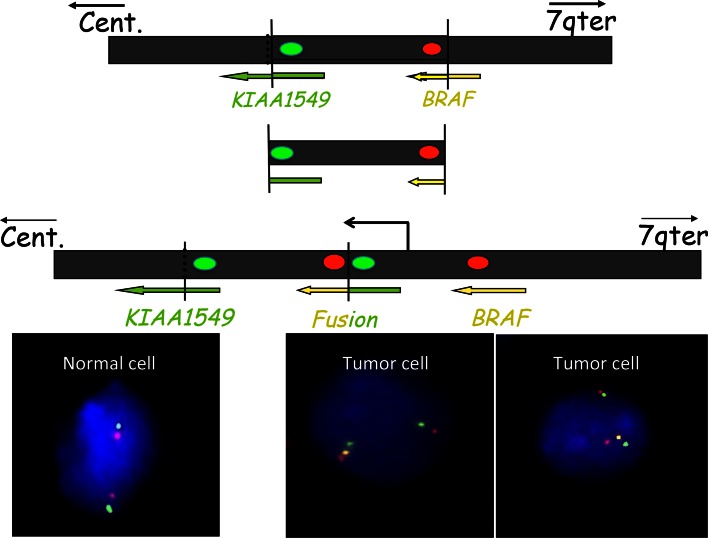
The common fusion rearrangement: The *upper black box* represents 7q34 on the long arm of chromosome 7. Both KIAA1549 and BRAF read towards the centromere (cent). A fragment of approximately 2 MB is duplicated and inserted at the breakpoint, producing a tandem duplication and the fusion between the 5′ end of KIAA1549 and the 3′ end of the BRAF gene that codes for the kinase domain. The fusion gene thus codes for the BRAF kinase domain together with the N-terminal part of KIAA1549, replacing the BRAF regulatory domain. It is important to remember that the exact breakpoints vary, resulting in nine combinations of KIAA1549-BRAF exons, all with an open reading frame from KIAA1549 spliced sequence into the BRAF sequence. This makes simple RT-PCR assays difficult. The *red* and *green* *dots* represent the location of FISH probes that could be used to identify the occurrence of the tandem duplication as demonstrated in the lower part of the figure, showing interphase normal and tumor nuclei with the tandem duplication hybridized with such probes. Note that the two unraveled normal chromosomes 7 in the normal nucleus show a *single* *red* and *green* *signal* adjacent to each other, while the tumor cell nuclei show one normal chromosomal signal but also a signal from the second chromosome 7, showing, in addition, a *yellow signal* (due to the fusion of the extra, now adjacent, *red* and *green signals*)

**Fig. 5:**
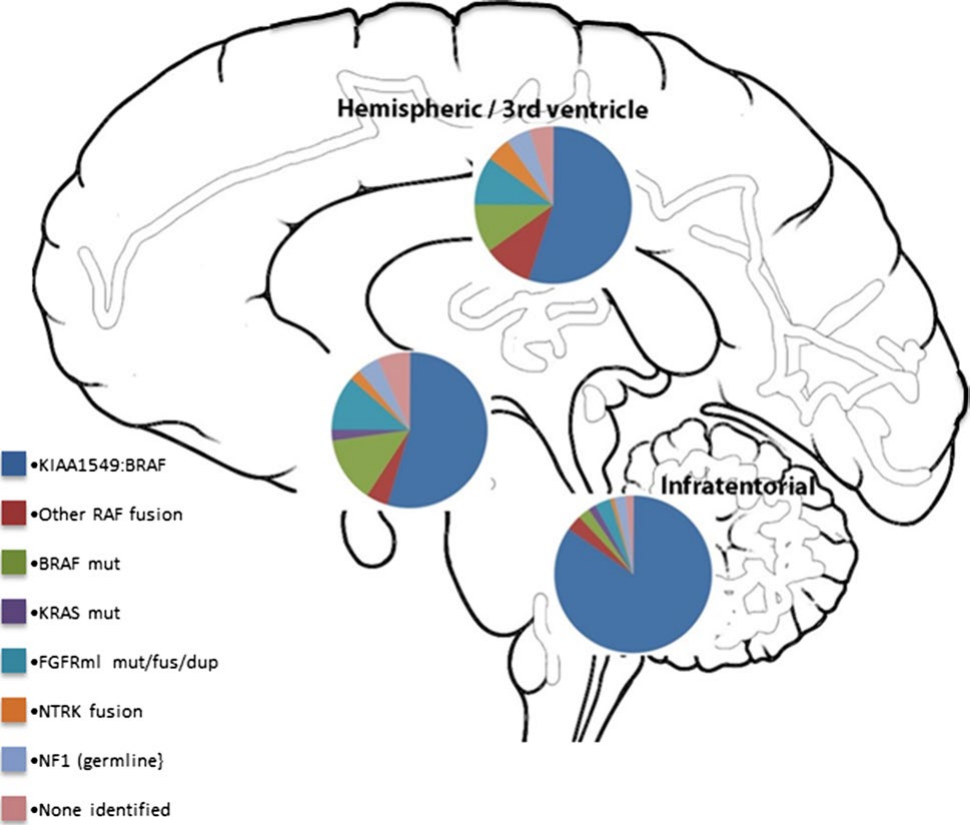
Pie charts summarizing the estimated frequency of particular MAPK pathway alterations in different anatomic locations (posterior fossa, diencephalon and cerebral hemispheres), calculated from a total of 188 PAs described in described in Zhang et al. [67] and Jones et al. [32]

**Fig. 6:**
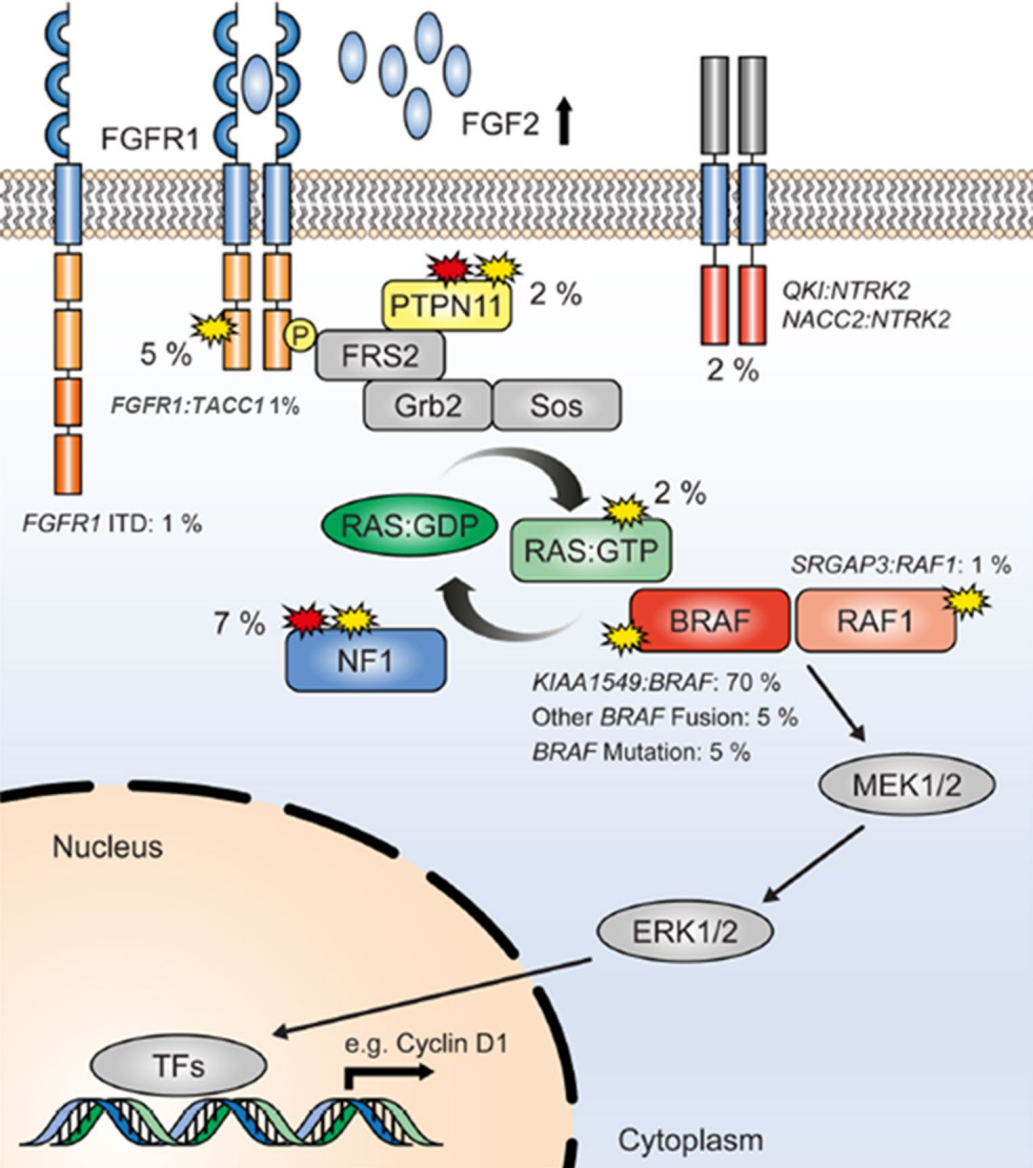
Summary showing the MAP kinase pathway with the approximate incidence of the different mutations in percent in a series of PAs (adapted with permission from Jones et al. [32])

Little was known about the genetics of PA until 2008. The only well-documented findings had been the association with NF1 syndrome and single reports of *KRAS* [30] or *PTEN* mutations [17] and the documentation of polysomy of chromosomes 5, 6, 7, 11, 15, and 20 by classical or array CGH that was almost exclusively found in older patients [33, 53]. In 2008, there were a number of publications documenting a commonly occurring 2 Mb duplication of 7q34, encompassing the *BRAF* gene in PAs [5, 16, 49]. This was rapidly recognized to be a tandem duplication, resulting in a transforming fusion gene between *KIAA1549* and *BRAF* (Fig. 4). The N-terminal end of the KIAA1549 protein replaces the N-terminal regulatory region of BRAF, while retaining the BRAF kinase domain that, being unregulated, becomes constitutively activated [21, 34, 59]. It was also recognized that, while the fusions between *KIAA1549* and *BRAF* overall were the most frequent genetic change in PAs (>70 %) and appeared to occur in almost all anatomical locations, they are most frequent in the cerebellar tumors and are less frequent at other sites (see Fig. 5) [5, 29] where mutations effecting other components of the MAPK pathway have been found [32, 67] (Fig. 6). Whole-genome sequencing of a substantial number of cases combined with RNA sequencing has shown that the average somatic mutation rate is very low in PAs, and almost all PAs studied in sufficient detail have been found to have mutations of genes coding for components of the MAP kinase pathway [32, 67]. Further, eight gene partners for *BRAF* fusions have been found in small numbers of cases (*FAM131B*, *RNF130*, *CLCN6*, *MKRN1*, *GNA11*, *QKI*, *FZR1 and MACF1*), all resulting in the loss of the N-terminal regulatory region of the BRAF protein and the retention of the kinase domain. These arise by various genetic mechanisms, including deletions and translocations [13, 21, 32, 67]. The commonly occurring BRAF fusions have been shown to be capable of transforming NIH-3T3 cells [34, 35]. Fusions between *SRGAP3* and *RAF1* have also been found in rare cases. As in the case of BRAF, the RAF1 fusion proteins retain the kinase domain of RAF and lose the regulatory domain with consequent constitutive activation and also activation of the MAP kinase pathway [21, 35, 67].

Identification of the *KIAA1549*-*BRAF* fusion has been used as a diagnostic marker for PAs, although there are some reports that this fusion may also occur rarely in adult diffuse astrocytic gliomas, including oligodendrogliomas together with loss of 1p and 19q and *IDH1* mutation [4]. Unfortunately, the fusions between *KIAA1549* and *BRAF*, while all results in an open reading frame and code for a fusion protein, including the BRAF kinase domain, can be derived from at least nine different fusion site combinations. This makes simple assays using, for example, PCR difficult if one wants to identify or exclude all variants of the fusion gene. This has resulted in FISH often being used to demonstrate the tandem duplication at 7q34 (Fig. 4), it being assumed to indicate the presence of a *KIAA1549*–*BRAF* fusion. The most common fusion is between *KIAA1549*-exon 16 and exon 9 of *BRAF*, followed by 15-9, and 16-11, and then by further rare exon combinations, including one which uses a cryptic *BRAF* acceptor splice site [32, 67].

Non-fusion mutations of the *BRAF* gene have also been identified in a subset of cases and include the well-known V600E mutation [21, 32, 35, 59] as well as a number of small insertions, which activate BRAF kinase signaling [32, 35].

The importance of the *NF1* gene in PAs associated with the NF1 syndrome has been shown with the inherited mutation being accompanied by a somatic mutation or loss of the patient’s single wild-type allele in the tumor cells [25]. As mentioned above, there is some evidence that germ-line mutations affecting the 5′ third of *NF1* gene may be associated with a greater chance of developing optic pathway Pas [7, 57], although this is yet to be confirmed in a large series.

The above alterations, identified in the era before next-generation sequencing, were together seen to account for the MAPK pathway “hit” in around 80–90 % of PAs. Two recent studies, however, have largely resolved the issue of which alterations define the remaining fraction [32, 67]. In addition to the rare BRAF fusion variants described above (with a growing list of 5′ partners), recurrent aberrations affecting the FGFR1 and NTRK family receptor kinases were observed. For the *NTRK* genes, these alterations were in the form of gene fusions, with the varying 5′ partners having a dimerization domain presumed to lead to constitutive dimerization and activation of the kinase [32, 67]. Interestingly, similar fusions were also recently described in a subset of infant high-grade gliomas [65]. For *FGFR1*, the changes were more varied, including hotspot point mutations (p.N546K, p.K656E); FGFR1–TACC1 fusions similar to those seen in adult GBM [61]; and a novel internal duplication of the kinase domain [termed TKD-duplicated or *FGFR1* internal tandem duplication (*FGFR1*-*ITD*)] [32, 67]. A summary of the incidence of all MAPK pathway alterations identified in PAs to date can be found in Fig. 6.

Interestingly, as indicated above, the spectrum of MAPK pathway alterations is not equal across all anatomic locations [5, 29, 32, 67]. The *KIAA1549*:*BRAF* fusion is extremely common in the cerebellum (found in approximately 90 % of cases) but somewhat less so supratentorially (although it is still very common). *FGFR1* alterations are largely restricted to midline structures, while BRAF V600E and NTRK family fusions are relatively more common in supratentorial tumors as a whole (summarized in Fig. 5). This variation is also observed in both the transcriptome and the methylome, with infratentorial tumors being distinguishable from their supratentorial counterparts on the basis of either gene expression or DNA methylation signatures [38, 58, 62]. The basis for this intriguing relationship between site/cell of origin and certain molecular alterations is currently unclear.

In the appropriate morphological context, the presence of these genetic alterations (particularly KIAA1549:BRAF fusion) can be used together with other findings to support the diagnosis of PA. It is clear, however, that the absence of such a fusion provides no diagnostic information, as there are so many other ways the MAP kinase pathway has been found to be activated in PAs. Unfortunately, many of the non-KIAA1549:BRAF alterations are also found to be similarly aberrant in the tumor types that are frequently among the differential diagnoses one has to consider when dealing with a difficult case. For example, the BRAF V600E mutation, while it occurs in a small subset of PAs, is a common mutation in gangliogliomas as well as pleomorphic xanthoastrocytomas and has also been reported in dysembryoplastic neuroepithelial tumors [12, 56]. This mutation can now be identified using a monoclonal antibody that specifically recognizes the substitution of glutamic acid (E) for valine (V) at position 600 in the BRAF protein [10]. A summary of genetic aberrations reported to date in PA, methods by which they may be identified and considerations as to their diagnostic utility, is given in Table 1. Notably, RNAseq is an extremely powerful method for detecting almost all classes of aberration observed in PA to date (with the possible exception of some NF1 alterations) and is, therefore, arguably the “gold standard” method of choice for molecular diagnostics in PA. At present, however, the cost and technical challenges (and requirement for fresh-frozen tissue) make this unfeasible in the majority of laboratories. A combination of other methods can be used, which will still provide valuable information, but it is unlikely that any (with the possible exception of full whole-genome sequencing) will be able to detect the entire range of changes observed. A further consideration when employing RNAseq is that high coverage is needed to detect some variants. For example, the KIAA1549:BRAF fusion is often expressed at relatively low levels and may be missed with insufficient sequencing depth (authors’ own observations).

**Table 1:** PA mutations, methods to detect them, and their diagnostic utility

MAPK pathway aberration	Preferred method	Alternative methods	Diagnostic utility
KIAA1549:BRAF	RNAseq	FISH (7q34 duplication); targeted RT-PCR (may miss some variants)	Highly recurrent in PA; extremely rare in other entities
Other BRAF/RAF1 fusions	RNAseq	NA (too many variants)	Recurrent in PA; extremely rare in other entities
BRAF V600E	Targeted sequencing	Anti-V600E IHC; WES; WGS; RNAseq	Recurrent in supratentorial PA; also common in GG/PXA/DNET
KRAS	Targeted sequencing (exons 2, 3)	WES; WGS; RNAseq	Rare in PA; frequency not fully established in other entities
FGFR1 mutation	Targeted sequencing (exons 12, 14)	WES; WGS; RNAseq	Recurrent in midline PA; frequency not fully established in other entities
FGFR1-ITD/fusion	RNAseq	WGS; targeted sequencing	Rare in PA; also observed in other LGG
NTRK fusions	RNAseq	NA (too many variants)	Recurrent in PA; also observed in other LGG and infant HGG
NF1	Clinical genetic testing	WGS; WES	Typically germline; closely associated with optic pathway PA

## Prognosis and treatment

In general, PAs are considered to have an excellent prognosis with overall 10-year survival reported to be over 90 % [9]. However, the prognosis for tumors in the hypothalamic/chiasmatic region (where the pilomyxoid variant occurs) and tumors where complete surgical resection was not carried out (or could not be carried out because of location) has less favorable progression-free and overall survival [14]. In addition, the occasional PAs that show leptomeningeal dissemination (not just localized leptomeningeal involvement alone) have a poorer outcome [14].

Pilocytic astrocytomas are primarily treated by surgery. This may be followed up by radiotherapy, particularly when there has been incomplete resection. Chemotherapy may be given in some cases where the tumor progresses, especially where further surgery is not possible.

Pilocytic astrocytomas only very seldom progress to a more malignant form, with the vast majority, even after multiple recurrences, maintaining their morphology and WHO grade I designation. However, small numbers of cases of apparent malignant transformation of a classical PA have been documented [1, 28, 36, 52].

The molecular findings have resulted in preliminary clinical trials of inhibitors of BRAF or targets further down the MAP kinase pathway. Initial studies with BRAF inhibitors have been published and demonstrated our incomplete understanding of the way the inhibitors affect the components of the MAP kinase pathway as well as the connections between this pathway and the PI3K/AKT/mTOR pathway. Sorafenib treatment of a small series of patients produced an unexpected acceleration of tumor growth irrespective of whether the tumor had a mutation of *NF1* or *BRAF*. In vitro studies have shown paradoxical activation of ERK by sorafenib in the context of the absence of wild-type NF1 [66], and the vemurafenib-related BRAF inhibitor PLX4720 has also shown paradoxical activation of the MAP kinase pathway in cells expressing the KIAA1549–BRAF fusion protein [60]. This may be explained by the fact that in BRAF/RAF dimers, where one of the two protomers has bound the drug, the non-drug-bound RAF becomes highly activated with increased ERK signaling (for a recent review of tumor responses to RAF family inhibitors see [41]). However, these therapies do appear to relatively effectively inhibit some types of tumors (at least initially) with the BRAF V600E mutation, and some second-generation inhibitors of BRAF have been reported that do not result in paradoxical activation of cell proliferation in cells expressing KIAA1549–BRAF fusion proteins [60].

## Differential diagnostic issues

While in its classic form and typical location PA can hardly be confused with any other CNS tumor, there are a number of situations in which making the diagnosis of PA may be challenging. The differential diagnosis among these tumors still largely relies on histopathological assessment. A finding of KIAA1549:BRAF fusion mutation and the absence of other changes, in association with the appropriate morphological features, is supportive of the diagnosis of PA. Unfortunately, most molecular markers cannot be used as definitive discriminatory findings as many have been reported to occur in primary brain tumors of various histogenesis in children and adults [2, 4, 12, 27, 37, 55].

The most frequent differential diagnosis of PA includes a number of “relatively circumscribed tumors” mostly of low grade (WHO grade I), including ganglioglioma (GG), dysembryoplastic neuroepithelial tumor (DNET), rosette-forming glioneuronal tumor of the fourth ventricle (RFGNT), but also pleomorphic xanthoastrocytoma (PXA) (WHO grade II). However, even glioblastoma can occasionally be considered.

Ganglioglioma is a grade I glioneuronal tumor composed of dysmorphic, frequently multinucleated ganglion cells accompanied by a glial component, resembling most often PA and less frequently either a diffuse astrocytoma or PXA. The number of neoplastic ganglion cells can be quite variable, and examples of truly “ganglion cell-poor” GG occur not infrequently. We have repeatedly encountered cases located in the temporal lobe (the most frequent single site of occurrence of GG) in which a first biopsy/resection showed a morphologic picture consistent with PA while a second resection, either to remove residual tumor or a tumor recurrence, demonstrated a definite GG. This has prompted us to often add a “disclaimer” to the diagnosis of PA in the temporal lobe “that PA is uncommon in the temporal lobe, and cases have occurred in which a second resection has disclosed a GG.” At the same time, in the cerebellum, GG should not be over-diagnosed, as large entrapped and distorted neurons from the deep nuclei can easily be mistaken for neoplastic ganglion cells. Pilocytic astrocytoma may mimic DNET with its typical oligodendroglial-like appearance; although frequently, the presence of glomeruloid-like vessels and the imaging appearance with enhancement point to the correct diagnosis.

**Fig. 7:**
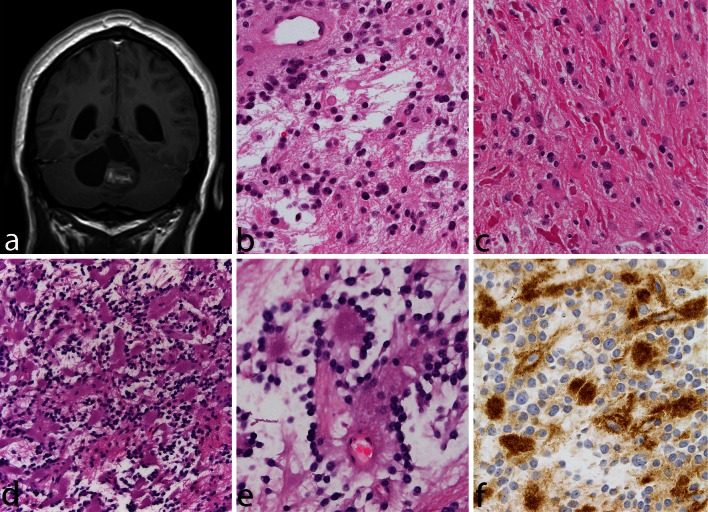
Pilocytic astrocytoma and rosette-forming glioneuronal tumor of the fourth ventricle. This solid and cystic tumor, occurring in a 26-year-old man, occupied the superior portion of the fourth ventricle (**a**). Most of the tumor displayed classic features of PA, being biphasic with microcystic areas (**b**) and areas with densely fibrillated tumors cells with abundant Rosenthal fibers (**c**). A small, distinct, very soft and * light gray* component was noted grossly, corresponding histologically to a classic “rosette-forming glioneuronal component” (**d**), with its characteristic high-power appearance (**e**) and synaptophysin positivity, corresponding to the center of the neuropil rosettes (**f**)

Rosette-forming glioneuronal tumor (RFGNT) is a very rare glioneuronal tumor, which occurs within the fourth ventricle region, affects preferentially young adults, and frequently shows histologic similarities with PA. We have encountered examples in which a small focus with features of RFGNT was present in an otherwise classic PA (Fig. 7). In contrast to PA, RFGNT have not been reported to harbor the *KIAA1549*–*BRAF* fusions or *BRAF* mutations. Recently, *FGFR1* mutations, previously described in PAs [32, 67] have been reported in 2 (of 8) RFGNT [23], indicating that in addition to histologic similarities, at least a subgroup of RFGNT may show molecular relationships with PA.

Pleomorphic xanthoastrocytoma, also a tumor of children and young adults, is typically a markedly cellular, epithelioid (rather than piloid) tumor. It is nearly always supratentorial, with only rare examples occurring in the cerebellum and spinal cord [27]. Unusual PA examples with hypercellularity and marked cellular pleomorphism may, however, be difficult to distinguish from PXA, a tumor with a much higher frequency of recurrence and more aggressive behavior than PA [27].

The most challenging and clinically most relevant differential diagnosis of PA, given the differences in prognosis and the treatment implications, is with diffuse gliomas of low and high grade. Histologically, PA may: lack its typical biphasic appearance; show a predominant oligodendroglial-like or astrocytic appearance without distinctive features; undergo acute hemorrhage, which may result in an “alarming” imaging appearance [64]; show necrosis and/or microvascular proliferation [24]. In our experience, these unusual features are most challenging when PA occurs in the supratentorial compartment, especially in adult patients, a group of patients and a location which would not readily prompt consideration of PA but rather of diffuse glioma. PA may closely mimic oligodendroglioma from which it can often be morphologically distinguished based on its relatively solid pattern of growth and presence (often only focal) of characteristic bipolar cells with Rosenthal fibers and/or eosinophilic granular bodies. In adults, the lack of *IDH1* or *IDH2* mutations, 1p19q co-deletion and/or presence of *BRAF* alterations are very helpful in sorting out the differential diagnosis, but this does not apply in children, in whom *IDH1* or *IDH2* mutations and/or 1p19q co-deletion are virtually absent. We have also seen supratentorial PAs, with marked cellular pleomorphism, recent hemorrhage and/or necrosis, and with prominent microvascular proliferation that have been mistaken for high-grade astrocytoma/glioblastoma. Critical examination of these cases has revealed a very low proliferative activity and lack of other features characteristic of a high-grade tumor; this has helped in reaching the correct diagnosis.

Piloid gliosis, a type of chronic gliosis characterized by a dense “refractile” fibrillary background with varying degrees of hypercellularity and glial atypia and associated with Rosenthal fibers, may be very difficult to distinguish from a monomorphous densely fibrillated PA. Piloid gliosis can be seen in a variety of conditions, including the wall of a spinal cord syrinx, adjacent to long-standing tumors such as craniopharyngioma or spinal ependymoma or in cyst walls, including pineal cysts. In a small biopsy from the spinal cord, a diagnosis of “dense piloid gliosis with Rosenthal fibers cannot exclude PA” may be all that is possible. Rarely, dense Rosenthal fiber deposition, often perivascular and subpial, may be indicative of Alexander disease, a rare genetic disorder caused by mutations in the glial fibrillary acidic protein (GFAP) gene, which may uncommonly manifest in children and/or adults with a spinal or brain stem mass-like lesion, prompting consideration of a neoplastic process and biopsy. The potential pitfalls we described will be magnified when only limited tissue is available, as can happen with small biopsies, common at certain sites (e.g., brain stem and spinal cord).

## Summary

We have come a long way in our understanding of the molecular changes that lie behind the development of PAs (and the other common pediatric brain tumors). The rate at which molecular findings have been incorporated into the diagnostic process at many centers has been impressive, but more remains to be done in this area. The molecular findings are complex, and the fact that the same mutations/rearrangements recognized to date can occur in many of the tumor types in our current classification and, in particular, are common to tumor types that can be included in the differential diagnosis of difficult cases where PA is being considered, makes their use demanding and difficult. At some point in the future, one can hope that specific patterns of multiple (both positive and negative) molecular findings will provide definitive diagnostic biomarker patterns. However, in the emerging era of personalized medicine and targeted therapies, “classification” arguably becomes less important as the molecular findings in the individual case, rather than a morphological grouping, will determine therapy. The ongoing updating of the WHO Classification of CNS tumors will ensure that the integration of molecular information into the neuropathological work-up of brain tumors becomes the norm [43]. While attempts at utilizing this knowledge in the treatment of children with PAs have, as yet, been unsuccessful, we await the results of ongoing trials targeting the MAP kinase pathway downstream of BRAF and with combination therapies. The huge advances in the last few years will undoubtedly continue, eventually leading to effective and specific individualized treatments of pediatric CNS tumors, including PAs that do not damage the surrounding developing brain.
